# The integrative panel of fatty acid desaturase-2 (FADS2) rs174583 gene polymorphism and dietary indices (DQI-I and HEI) affects cardiovascular risk factors among obese individuals

**DOI:** 10.1186/s12902-023-01289-3

**Published:** 2023-02-15

**Authors:** Mahsa Mahmoudinezhad, Sheida Khosravaniardakani, Leila Saljoughi Badelou, Ehsan Fayyazishishavan, Houman Kahroba, Mahdieh Abbasalizad Farhangi

**Affiliations:** 1 Molecular Medicine Research Center, Tabriz University of Medical Sciences, Tabriz, Iran; 2 Department of Epidemiology, Robert Stempel College of Public Health, Florida International University, 1240 S.W.108 AVE, Path, University Park, Miami, FL 33174 USA; 3 Department of Internal Medicine, School of Medicine, Shahid Beheshti University of Medical Sciences, Tehran, Iran; 4 Department of Biostatistics and Data Science, School of Public Health, The University of Texas Health Science Center at Houston (UTHealth), Houston, TX 77030 USA; 5 Department of Toxicogenomics, GROW School of Oncology and Development Biology, Maastricht University, Maastricht, the Netherlands; 6 Centre for Environmental Sciences, Hasselt University, Hasselt, Belgium; 7 Drug Applied Research Center, Tabriz University of Medical Sciences, Attar-neishabouri Ave, Golgasht St, Tabriz, 5165665931 Iran; 8 Department of Community Nutrition, Faculty of Nutrition, Tabriz University of Medical Sciences, Tabriz, Iran

**Keywords:** Fatty acid desaturase, Diet quality index-international, Healthy eating index, Obesity

## Abstract

**Background:**

Recent studies have shown that dietary intakes and gene variants have a critical role in the obesity related comorbidities. This study aimed to evaluate the effects of the interactions between Fatty acid desaturase 2 (FADS2) gene rs174583 polymorphism and two dietary indices on cardiometabolic risk factors.

**Methods:**

This cross-sectional study was carried out on 347 obese adults aged 20-50 years old in Tabriz, Iran. Healthy eating index (HEI) and Diet quality index-international (DQI-I) were evaluated by a validated semi-quantitative 147-item Food frequency questionnaire (FFQ). Polymerase chain reaction-restriction fragment length polymorphism (PCR-RFLP) was used to determine FADS2 gene variants. Multivariate analysis of covariance (MANCOVA) was used to identify gene-diet interactions on metabolic parameters.

**Results:**

Waist circumference (WC) and serum triglyceride (TG) levels were significantly higher among carriers of TT genotype of FADS2 gene (*P* < 0.05). In addition, the interactions between FADS2 gene rs174583 polymorphism and DQI-I had significant effects on weight (P _interaction_ = 0.01), fat mass (P _interaction_ = 0.04), fat free mass (P _interaction_ = 0.03), and Body mass index (BMI) (P _interaction_ = 0.02); the highest level of these parameters belonged to TT carriers. Similarly, the interactions between FADS2 gene variants and HEI had significant effects on insulin (P _interaction_ < 0.001), Homeostasis model assessment of insulin resistance (HOMA-IR) (P _interaction_ < 0.001), Quantitative insulin check index (QUICKI) (P _interaction_ = 0.001), and alpha Melanocyte stimulating hormone (α-MSH) (P _interaction_ = 0.03).

**Conclusion:**

In this study, for the first time, we reported the effects of gene-diet interactions on metabolic traits. Compliance with dietary indices (DQI-I and HEI) ameliorated the adverse effects of gene variants on metabolic risk factors, especially in heterogeneous genotypes. Further prospective cohort studies are needed to confirm these results.

**Supplementary Information:**

The online version contains supplementary material available at 10.1186/s12902-023-01289-3.

## Background

The prevalence of obesity has been doubled since 1980 and now accounts for one-third of the world’s population [1, 2]. This ascending trend in the prevalence of obesity across the globe is true in Iranian population too. Likewise, the prevalence of obesity was estimated to be 21.7% in Iran, which demands urgent actions [3]. Obesity is a multifactorial disorder which contributes to increased risk of chronic diseases such as type two diabetes mellitus (T2DM), cancers, and cardiovascular disease (CVD); it has also negative effects on life expectancy, quality of life, and health care costs [1, 4–6]. Obesity is characterized by excessive body fat accumulation due to increased energy intake and decreased physical activity [7–10]. Although it is internationally accepted to use Body mass index (BMI) to classify the obesity status, it is not an accurate index for body fat distribution and body composition [1, 11].

Despite significant progress to overcome the current health problem and the need to transit from basic nutritional science to clinically relevant dietary recommendations, nutrigenomics and nutrigenetics seem to be challenging to fill the gap in clinical nutrition. This addresses the substantial between-individual variability in response to dietary interventions in a molecular and metabolic level and considers the gene-diet interactions [12].

**Fig. 1:**
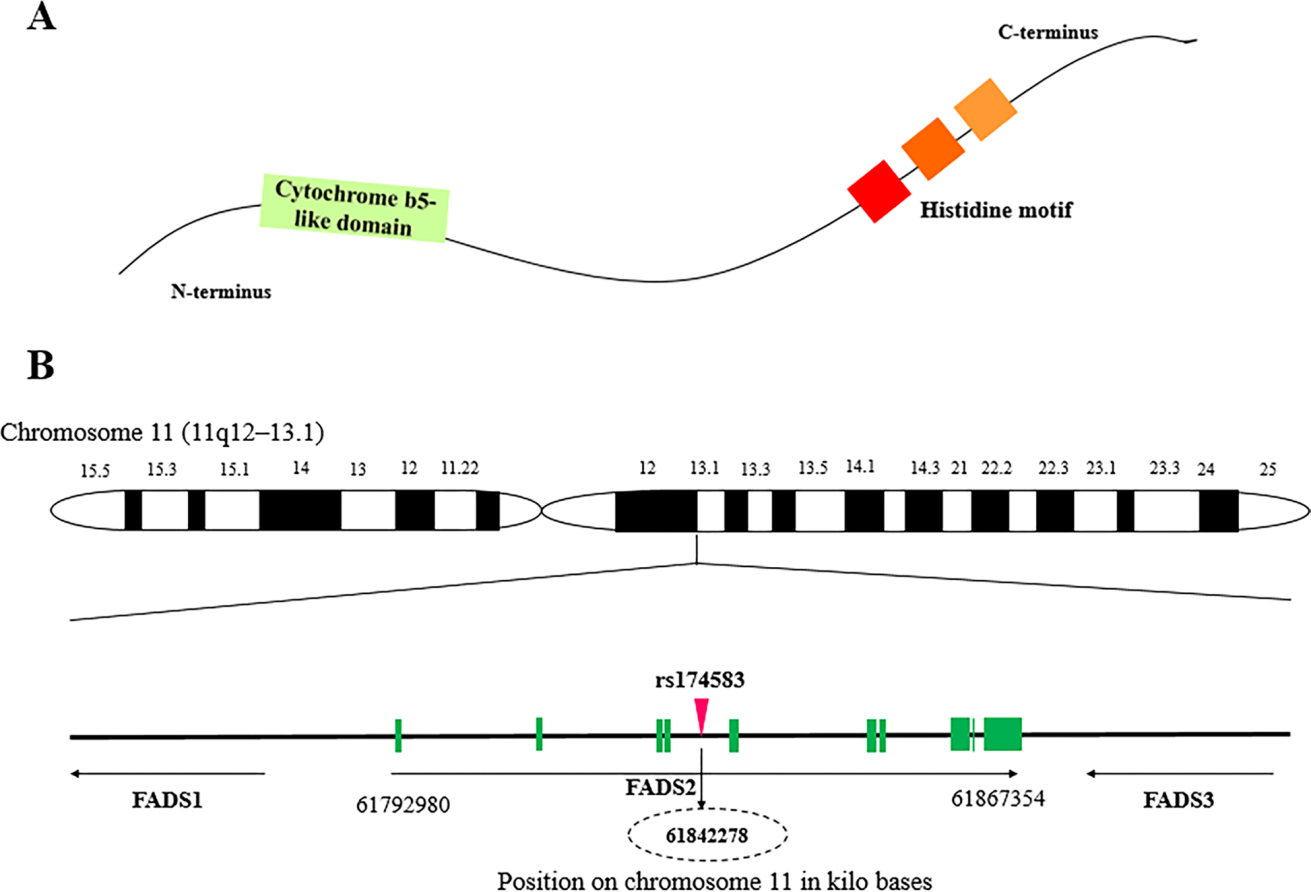
Schematic representation of FADS (**A**) and the human FADS gene cluster located on chromosome 11 with exon/intron organization (**B**)

**Fig. 2:**
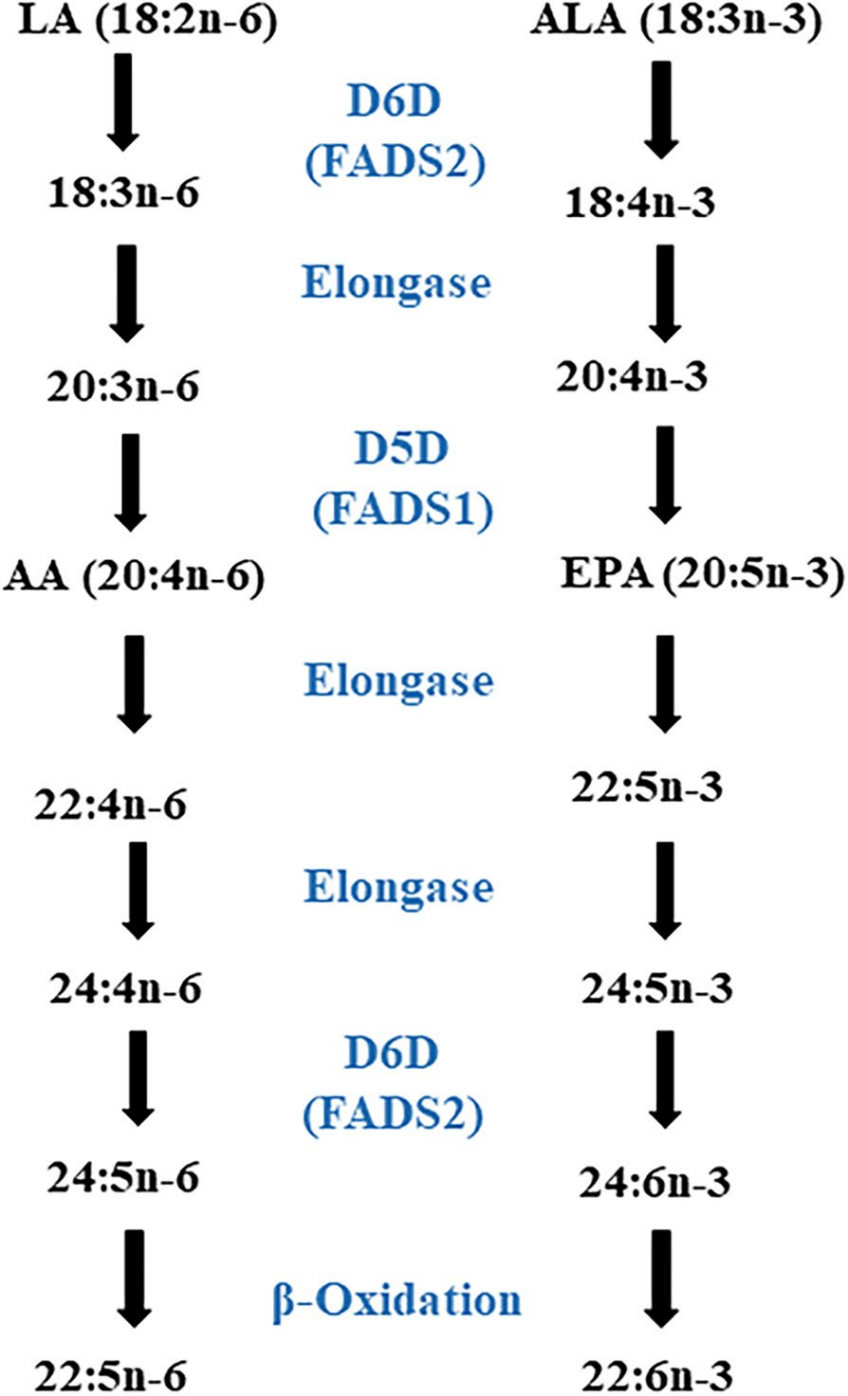
Synthetic pathways of long chain poly unsaturated fatty acids

The development of genome studies have generated opportunities to design a personalized nutrition based on genetic makeup in population [13]. Genome wide association studies (GWAS) have paved the way for identification of Single nucleotide polymorphisms (SNPs) as drivers of cardiometabolic diseases. Therefore, SNPs have been known as a proper choice to identify phenotypic differences and address inter-individual variability [14]. In this regard, it is of great importance to identify SNP in the fatty acid desaturase (FADS) genes and determine their interaction with environmental factors such as diet in relation to risk of metabolic diseases. FADS1 and FADS2 appear side-by-side on chromosome 11 and therefore a degree of overlap may exist and it has been shown that SNP of rs174547 in FADS1 and rs174583 in FADS2 are in high linkage disequilibrium [15]. However, it has been shown that FADS2 gene rs174583 polymorphism is involved in fatty acids metabolism [16]. FADS2 is an endoplasmic reticulum membrane-bound protein located on chromosome 11 (11q12–13.1) from 61,792,980 to 61,867,354 in forward strand, which is comprised of 12 exons and 11 introns and rs174583 is located in 61,842,278 kb [17–20]. FADS2 has the desaturation domain on the C-terminal with histidine rich region and a cytochrome b5 domain in N-terminal position (Fig. 1) [19]. It also encodes delta 6 desaturase (D6D) as a rate limiting enzyme in the synthesis of Long chain poly unsaturated fatty acids (LC-PUFAs) from essential fatty acids [17, 21, 22]. Since w-3 and w-6 pathways share the same enzymes for desaturation and elongation, D6D is involved in the desaturation of dietary obtained linoleic acid (LA) and alpha linolenic acid (α-ALA) to gamma linoleic acid (γ-ALA) and stearidonic acid, respectively [19]. Arachidonic acid (AA) and eicosapentaenoic acid (EPA), produced in the LA and ALA pathways respectively, are affected by elongase and D6D; this facilitates the production of docosahexaenoic acid (DHA) in n-3 pathway subsequently [19, 21]. Although initial steps of LC-PUFA biosynthesis are performed in endoplasmic reticulum, DHA production is conducted in the peroxisome where β-oxidation of LC-PUFAs takes place (Fig. 2) [23, 24]. Although knowledge about the effect of FADS2 gene polymorphism and their potential role in the development of metabolic diseases is limited. However, several studies have pointed to the association of FADS polymorphism with AA which is known as a precursor of low-grade inflammation and it will facilitate the condition accompanied with lipid metabolism disorders including obesity, CVDs, T2DM and non-alcoholic fatty liver disease [25–27]. PUFAs act as biological ligands to functional pathways and modulate the function of membrane lipid rafts, so fatty acid composition may alter metabolic function [26]. Also, PUFAs are involved in immunometabolic responses and the pathogenesis of chronic diseases associated with lipid metabolism disorders [28]. In addition, some studies have illustrated the effects of FADS2 gene polymorphism in glucose hemostasis too [25]. While, Mazoochian et al. showed no significant differences about the distribution of rs174583 genotypes between diabetic patients and control groups [29].

Previous studies have suggested that dietary intakes may interact with FADS2 gene rs174583 polymorphism to modify cardiometabolic factors in obese individuals [17, 29, 30]. So far, several dietary approaches have been developed. However, since the nutrients are not used in isolation as an individual dietary component, multidimensional method is preferred to single nutrient assessment to provide a comprehensive diet quality assessment [31]. Healthy eating index (HEI) and Diet quality index-international (DQI-I) may provide a broader and more realistic image of dietary intake by exerting cumulative and interactive effects of nutrients [32, 33] and help to clarify the gene-diet interactions. Therefore, our primary outcome was to evaluate metabolic factors among different FADS2 genotypes. Accordingly, for the first time, we evaluated the effects of the interactions between FADS2 gene rs174583 polymorphism and two dietary indices (HEI and DQI-I) on cardiometabolic risk factors among obese individuals as a secondary outcome.

## Methods

### Study population

This cross-sectional study included 347 obese individuals. The samples were obtained from two separate projects including 188 and 159 obese participants in Tabriz, Iran. The inclusion criteria were as follows: age range of 20-50 years, BMI range of 30-40 kg/m^2^, with no history of alcohol or drug abuse, and signing a written informed consent. We also excluded the subjects with a history or presence of chronic diseases (such as T2DM, cardiovascular diseases, cancers, kidney diseases, and infections), individuals reporting any possible changes in their usual diet, pregnant or lactating women, and those using drugs affecting weight such as corticosteroids. The sample size calculation, using G-power software was based on the r of 0.25, α = 0.05 and β = 0.20, and power 80%, the minimum sample size was 290 and considering 15% drop-out, the final sample size was 347 individuals. The statistical power of 80% was used to avoid false negative associations and to determine a cost-effective sample size. Therefore, we decided to subgroup the individuals into tertiles for maintaining the power of 80% and also for assigning the comparable number of participants to each group accordingly.

The study protocol was fully explained to all participants and a written informed consent was obtained from them. The protocols of both projects had already been approved by the ethics committee of Tabriz University of Medical Science, Tabriz (IR.TBZMED.REC.1399.062. and IR.TBZMED.REC.1398.460).

### Anthropometric measurements

First, the demographic data of participants was collected by interviewing. Additional data on socioeconomic status (SES), including educational status, occupation, marital status, and family size was obtained and the subjects were classified into three tertiles (low, middle, and high) according to the total scores. The International Physical Activity Questionnaire (IPAQ) was used to investigate the physical activity status in subjects [34]. Weight assessment was performed by calibration of the Seca scale (Seca, Germany) in standing unassisted position with light clothing and no shoes to the nearest 0.1 kg. Height was measured by standing on heels against the corner where the wall and floor meet considering head, shoulders, and buttocks touching the wall by a stadiometer near to 0.5 cm. BMI was calculated according the Quetelet’s formula [4]. Waist circumference (WC) was measured with a non-stretchable tape around the middle and above the umbilicus without compressing the skin to the nearest 0.1 cm over light clothing. Hip circumference (HC) was measured according to the widest part of buttocks. In addition, bioelectrical impedance analysis (BIA) was used to estimate body composition. Systolic blood pressure (SBP) and diastolic blood pressure (DBP) were measured in a relaxing position with mercury sphygmomanometer.

### Measurements of biochemical parameters

Fasting blood samples (10 ml) were obtained after a 12-hour overnight fasting. Serum and plasma isolations were done using centrifugation at 4500 rpm at 4 °C for 10 min immediately. Then, aliquots were frozen at − 80 °C until use. Total cholesterol (TC), high-density lipoprotein cholesterol (HDL-C), triglyceride (TG), and serum glucose concentrations were measured using a commercial kit (Pars Azmoon, Tehran, Iran). Serum low-density lipoprotein cholesterol (LDL-C) was calculated by Friedewald’s equation using TC, TG, and HDL-C concentrations [35]. In addition, enzyme-linked immunosorbent assay (ELISA) kits were used to detect insulin levels. Homeostasis Model Assessment-Insulin Resistance Index (HOMA-IR) and Quantitative Insulin Sensitivity Check Index (QUICKI) were calculated according to the specified formula [36, 37]. Moreover, plasma Agouti-Related Peptide (Ag-RP) and α-Melanocyte-Stimulating Hormone (α-MSH) were assayed using ELISA kits (Bioassay Technology Laboratory, China).

### Appetite assessment

Appetite was measured by Visual Analogue Scale (VAS) (100-mm line) to record the feelings about hunger, satiety, fullness, and desire to eat salty, sweet, or fatty foods. This was done by making a mark on a 100-mm line with ‘I’m not hungry at all’ and ‘I have not been so hungry’ at tow ends. The distance from the left end up to marked one was considered as appetite score [38].

### Dietary assessment

A trained dietitian estimated the usual dietary intakes of participants using a validated semi-quantitative Food Frequency Questionnaire (FFQ) with 147 food items [39, 40]. The questions were explained to all subjects, and they were asked to report the frequency (daily, weekly, monthly, or yearly) and amount of each food item. Afterward, all these responses were changed to gram using household measurements [41]. Accordingly, nutrient and energy intakes were calculated using Food Composition Table (FCT).

DQI-I was designed to capture and represent both food and nutrient intake together. The DQI-I, as a healthy diet index, is based on four factors, including variety, adequacy, moderation, and balance to resolve all nutritional concerns. Each item has its own score as follows: the range of dietary variety score is 0-15 for overall dietary diversity and 0-5 for a protein source (fish, meat, poultry, dairy, eggs, and beans); adequacy scores range between 0 and 40 according to the amount of protein, vegetable, fiber, fruit, grain, calcium, iron, and vitamin C intake to ensure consumption of a healthy diet; and moderation scores such as saturated fat, cholesterol, total fat, sodium, and junk foods get 30 and 10 points, allocated to the overall balance based on fatty acid and macronutrient ratio. Finally, the total DQI-I score constitutes the sum of these categories and higher DQI-I level indicates better diet quality [42].

HEI-2015 was developed to demonstrate the compliance of dietary intakes with Dietary Guidelines for Americans (DGA). HEI constitutes of 13 components (four moderation and nine adequacy), which are scored separately; total score of HEI is derived from the sum of each component. Three adequacy components (e.g., dairy, whole grains, and fatty acids) score between 0 and 10, where a higher score indicates a higher intake. Other six adequacy components, including total fruits (canned fruit, fruit, and fruit juice), whole fruits (fruits except fruit juice), plant proteins, total vegetables, greens, seafood, beans, and total protein score 5 for the highest intake and 0 for the poorest intake. Also, higher intake of four moderation components such as refined grains, added sugars, sodium, and saturated fats reflect lower intakes [43].

### DNA extraction and genetic sequencing

Single nucleotide polymorphism (SNP) was chosen from studies claimed to be associated with obesity [29] and according to minor allele frequency from previous studies [44]. Chloroform technique was used to extract genomic DNA from whole blood. In this technique the cell lysis buffer was used to remove proteins binding to DNA and membrane of red blood cell. Likewise, chloroform was used to remove the remaining proteins. Accordingly, three phases were formed in the micro tubes that the upper phase contained DNA.

**Fig. 3:**
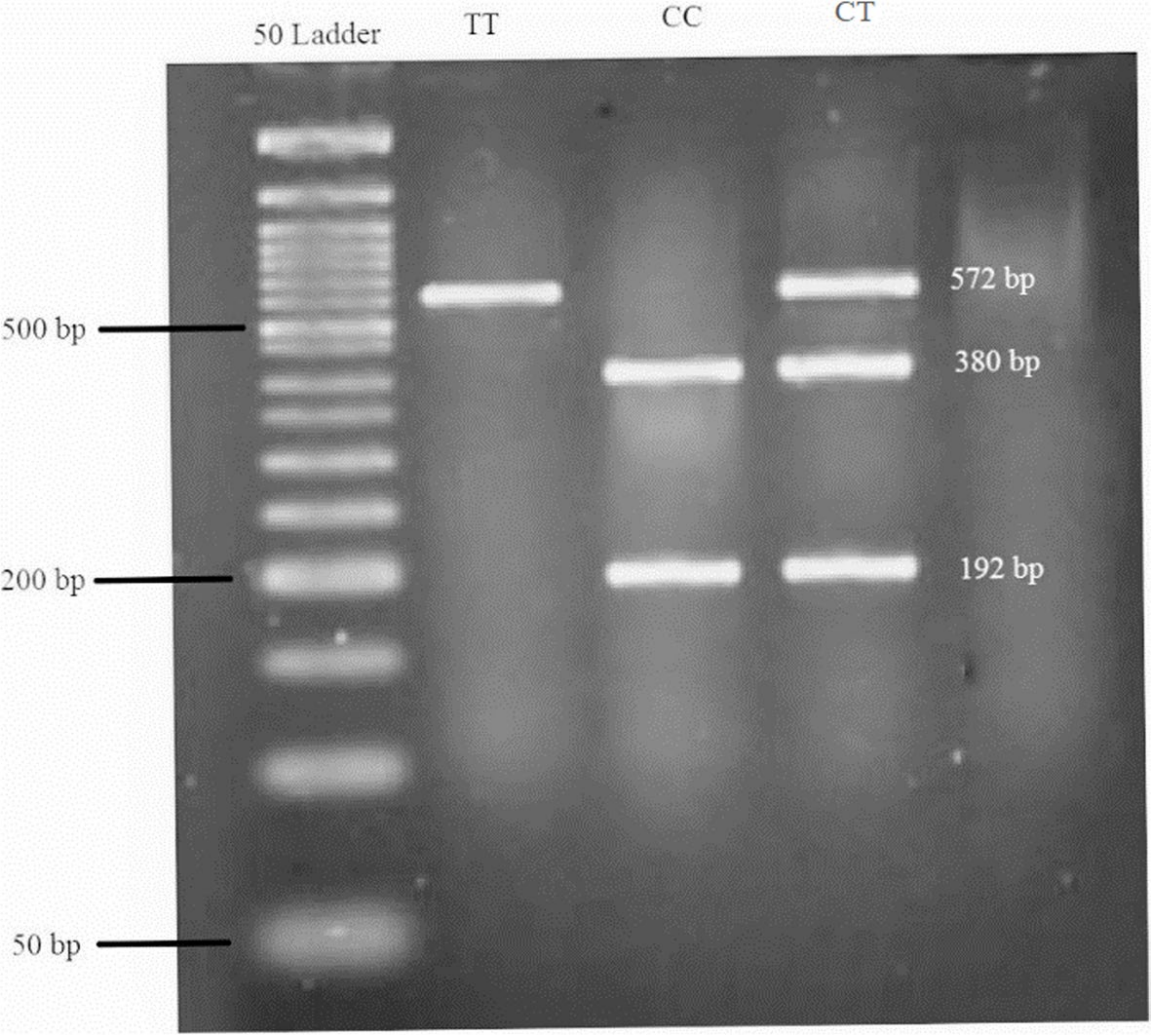
PCR-RFLP analysis of genotyping for the FADS2 rs174583 variants by TauI. Length of the digested products were determined by 50-bp ladder. TT = homozygous mutated (572 bp), CC = homozygous wild-type (380 and 192 bp), CT = heterozygous mutated (572, 380, and 192 bp)

The concentration of extracted DNA was checked using Nano Drop ND-1000 spectrophotometer. Likewise, all available DNAs were expected to be genotyped for rs174583. Then, polymerase chain reaction-restricted length polymorphism (PCR–RFLP) method was carried out to determine genotypes of SNP rs174583 located in the 61,842,278 position of chromosome 11 in the intron of FADS2 (Fig. 3). The forward and reverse primers were 5′ AGGAAGCAGACCACAGAGTC 3′ and 5′ TCCTTCGTCTGGTGTCTCAG 3′, respectively. PCR reactions were performed in a final volume of 10 μl containing 2 μl of extracted DNA, 5 μl of Master Mix (Ampliqon; Denmark), 2 μl of distilled water, and 1 μl of primers. The PCR cycles in a DNA thermocycler (BIO RAD T100 Thermal Cycler) were optimized to 10 min of denaturation at 95 °C; amplification consisted of 35 cycles at 94 °C; annealing at 60 °C for 20 s, 50 s of extension at 74 °C, and final extension for 10 min at 74 °C. In addition, TauI (cat. Num ER1652, USA), as a restriction enzyme, was used to digest amplified DNA. The enzymatic digestion with a final volume of 20 μl containing the PCR product, TauI enzyme and buffer was performed at 56 °C. Three possible genotypes of FADS2 rs174583 were detected as follows: homozygous mutated TT (572 bp), homozygous wild-type CC (380 and 192 bp), and heterozygous mutated CT (572, 380, and 192 bp). Finally, all the digested PCR products were visualized by green stained gel of electrophoresis on 1.5% agarose gel in a Gel Doc-system (U.V.P Company, Cambridge, UK).

### Statistical analysis

Data were analyzed using SPSS software (SPSS Inc., USA, version 25). Normality of variables was checked according to mean, standard deviation (SD), skewness, and kurtosis. Normal and non-normal data were expressed as mean ± SD and median (min, max), respectively. Meanwhile, categorical values were reported as frequency (%) using χ^2^ test. Chi-square and independent sample t-test tests were used to compare qualitative and quantitative variables, respectively. Multivariate analysis of covariance (MANCOVA) was used to investigate the effect of interactions between FADS2 rs174583 polymorphism and diet quality indices on cardiometabolic risk factors. *P*-values less than 0.05 were considered as statistically significant.

## Results

This cross-sectional study was conducted among 347 obese individuals (58.2% males vs. 41.8% females). The demographic, clinical, and biochemical characteristics of participants are presented in Table 1. The mean (SD) age, weight, and BMI were 40.78 ± 9.23 years, 92.11 ± 14.44 kg, and 32.62 ± 4.80 kg/m^2^, respectively. As can be seen, the majority of participants (47.9%) had low physical activity with middle SES (52.90%). Genotype distribution was as follows: CC (37.8%), CT (51.9%), and TT (10.3%).

**Table 1:** General characteristics and biochemical parameters of study participants

	**(%)**
Sex (%)	
Men	58.20
Women	41.80
SES (%)	
Low	2.70
Middle	52.90
High	44.4
Physical activity level (%)	
Low	47.90
Moderate	27.70
High	24.50
	**Mean (SD) or Median** **(25 and 75 percentiles)**
Age (years)	40.78 ± 9.23
Weight (kg)	92.11 ± 14.44
BMI (kg/m^2^)	32.62 ± 4.80
FM (kg)	33.81 ± 9.13
FFM (kg)	62.25 ± 12.35
WC (cm)	106.78 ± 9.62
WHR	0.93 ± 0.07
Appetite	33.58 ± 8.93
SBP (mmHg)	122.99 ± 16.35
DBP (mmHg)	81.78 ± 11.69
TC (mg/dl)	191.45 ± 36.64
HDL (mg/dl)	43.32 ± 9.52
LDL(mg/dl)	123.68 ± 31.83
TG (mg/dl)	119.97 ± 58.46
Glucose (mg/dl)	90.00 (83.75, 98.00)
HOMA-IR	2.91 (1.87, 4.93)
QUICKI	0.32 ± 0.03
Insulin (U/ml)	13.00 (8.60, 21.12)
Ag-RP (Pg/ml)	23.95 (19.60, 30.97)
α-MSH (ng/L)	146.50 (130.50, 186.80)

*SES* Socio-economic status, *BMI* Body mass index, *FM* Fat mass, *FFM* Fat free mass, *WC* Waist circumference, *HC* Hip circumference, *SBP* Systolic blood pressure, *DBP* Diastolic blood pressure, *TC* Total cholesterol, *HDL* High-density lipoprotein, *LDL-C* Low density lipoprotein cholesterol, *TG* Triglyceride, *HOMA-IR* Homeostasis model assessment of insulin resistance, *QUICKI* Quantitative insulin sensitivity check index, *AgRP* Agouti-related protein, *α-MSH* Alpha melanocyte stimulating hormone

**Fig. 4:**
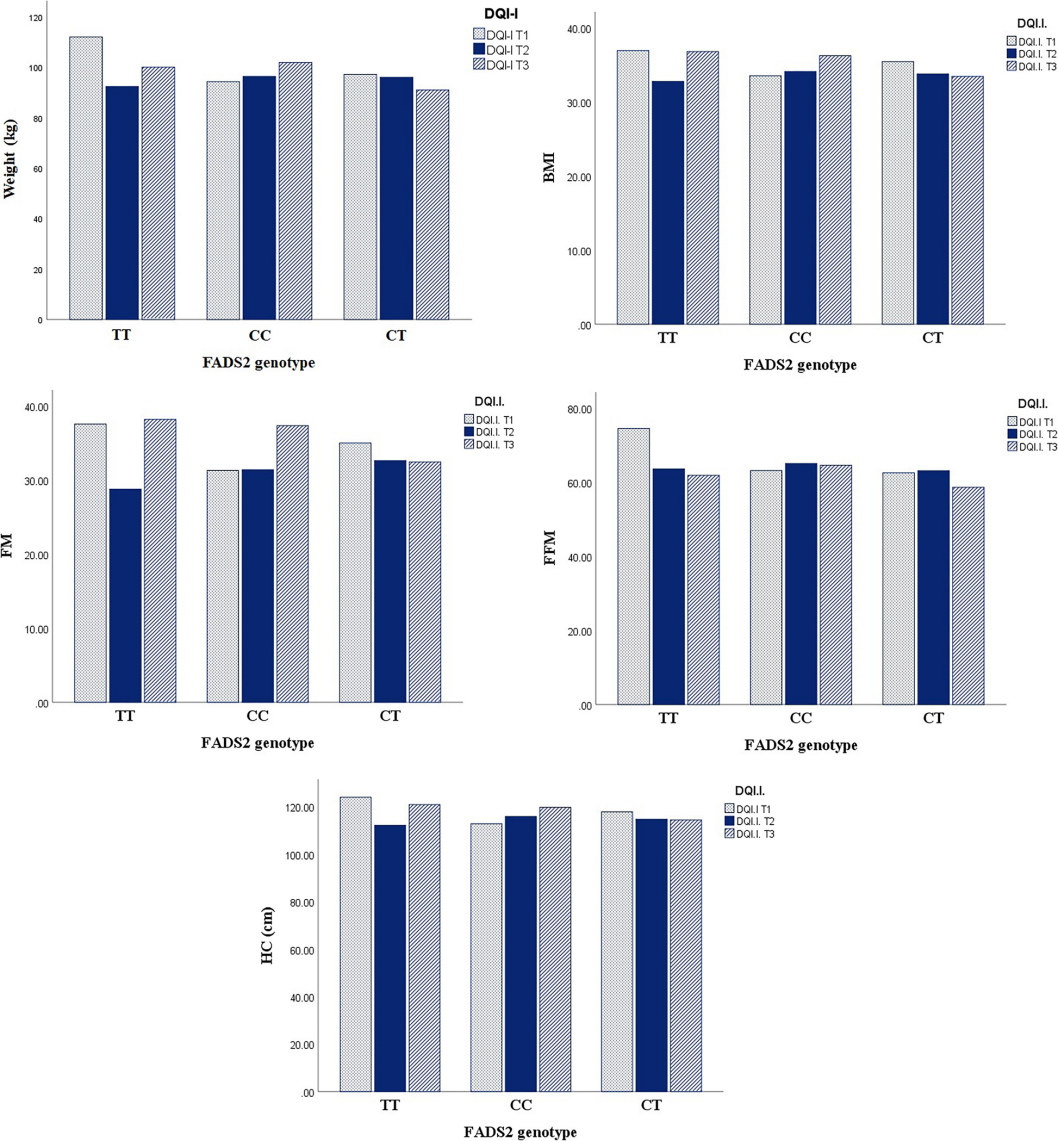
FADS2 genotypes interact with different adherence to DQI-I in relation to cardio-metabolic factors

**Fig. 5:**
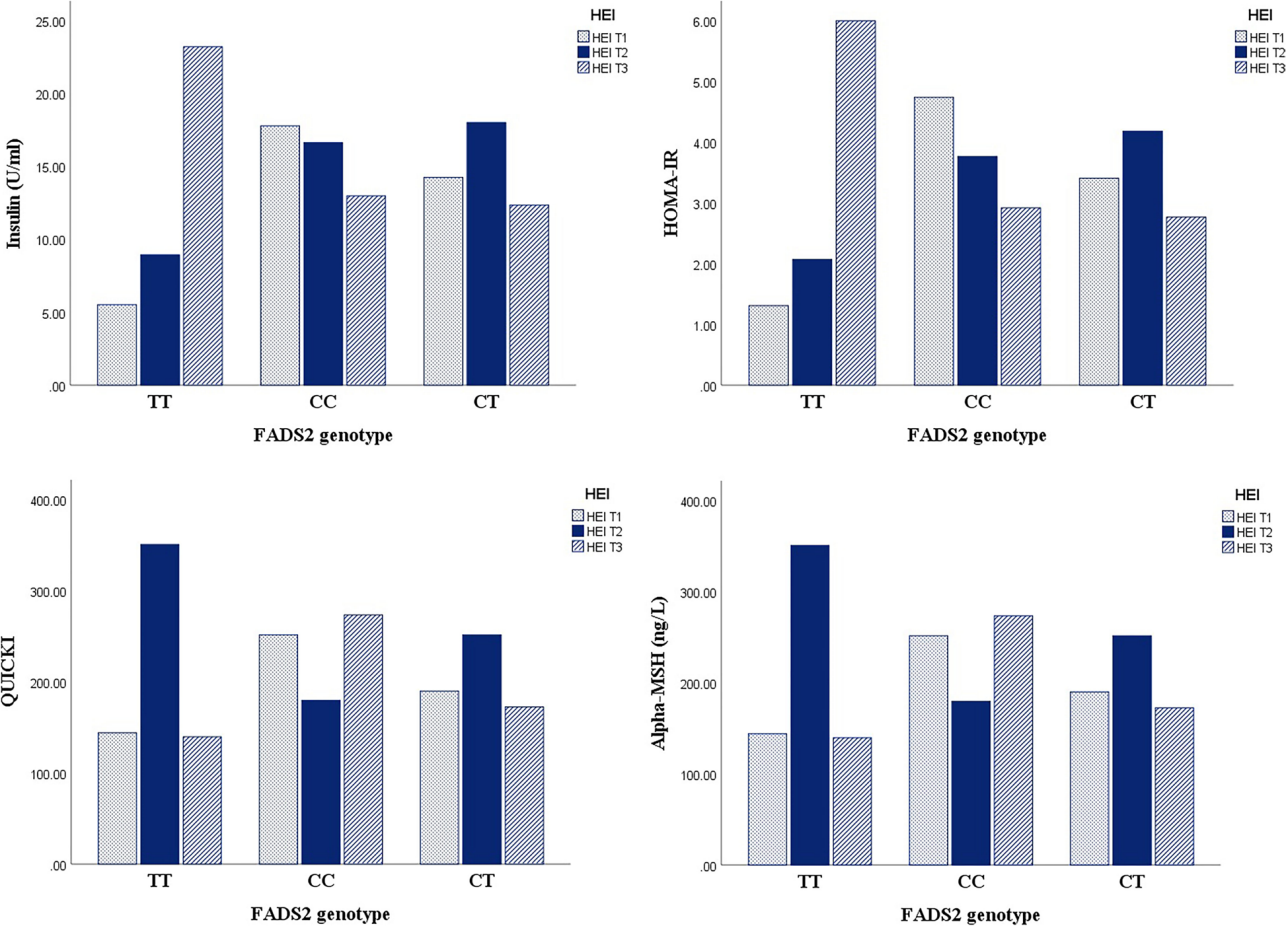
FADS2 genotypes interact with different adherence to HEI in relation to cardio-metabolic factors

The comparison of anthropometric and biochemical parameters across genotypes of FADS2 is presented in Table 2. As the table shows, there were significant differences in WHR (*P* = 0.04), WC (P = 0.04), and TG (*P* = 0.03) levels between FADS2 genotypes. Moreover, adults with adverse lipid profile were more likely to have TT carriers. However, there was no significant differences between genotypes except for TG value. Also, TT carriers had higher levels of anthropometric values, including weight, BMI, WC, and fat mass. Also, dietary vitamin are compared between FADS2 genotype and are provided in supplementary table 1. In addition, Table 3 presents analysis for the association between biochemical values and FADS2 rs174583 genotypes. There was no significant association between dietary biochemical values and FADS2 rs174583 genotypes in either crude or multivariate-adjusted model except for HDL value. Furthermore, there were significant interactions between FADS2 gene rs174583 polymorphism and DQI-I in terms of weight (*P* = 0.01), FM (*P* = 0.04), FFM (*P* = 0.03), BMI (*P* = 0.02), and HC (*P* = 0.005) (Fig. 4) even after adjusting for potential confounders such as age and sex. We witnessed higher levels of weight, BMI, FM, FFM, and HC in subjects with lower adherence to DQI-I diet in TT genotype. In contrast, by increasing adherence to DQI-I, we observed the downward trend for these parameters in this genotype compared to the first tertile of DQI-I, indicating that DQI-I may modify the adverse effects of having TT genotype. Moreover, insulin (*P* < 0.001), QUICKI (*P* = 0.001), HOMA-IR (P < 0.001), and α-MSH (*P* = 0.03) had a significant interaction with FADS2 genotypes and HEI in obese adults (Fig. 5). With respect to gene-diet interactions, the highest level of insulin and HOMA-IR were seen in TT carriers even in those with high compliance to HEI. This shows that TT carriers may experience high levels of insulin and HOMA-IR irrespective of having high-quality diet. All other gene-diet interactions (gen-DQI-I and gene HEI) in term of biochemical values are provided in supplementary Tables 2 and 3.

**Table 2:** Comparison of anthropometric and biochemical values across FADS_2_ gene rs174583 polymorphism

Variables	Genotype			P^*^
	CC	CT	TT	
Genotype distribution (%)	37.8	51.9	10.3	0.40
Age (year)	38.38 ± 7.39	37.23 ± 7.15	39.43 ± 6.62	0.43
Weight (kg)	97.31 ± 13.61	94.79 ± 13.12	99.05 ± 12.27	0.35
BMI (kg/m^2^)	34.53 ± 3.56	34.25 ± 3.91	35.51 ± 4.02	0.47
WC (cm)	110.57 ± 8.62	106.71 ± 10.31	110.87 ± 10.36	**0.04**
WHR	0.93 ± 0.06	0.92 ± 0.07	0.93 ± 0.07	**0.04**
FM (%)	33.22 ± 8.95	33.43 ± 9.18	35.12 ± 9.08	0.75
SBP (mmHg)	113.67 ± 19.72	115.59 ± 13.01	116.93 ± 12.51	0.68
DBP (mmHg)	75.83 ± 13.81	76.79 ± 11.75	74.56 ± 8.74	0.77
**TC (mg/dL)**	183.62 ± 29.77	189.04 ± 35.76	193.12 ± 32.50	**0.49**
**HDL (mg/dL)**	44.72 ± 8.65	46.35 ± 8.08	40.81 ± 9.94	**0.05**
**LDL (mg/dL)**	115.50 ± 27.12	119.58 ± 32.94	121.45 ± 29.37	**0.66**
**TG (mg/dL)**	102.00 (33.00, 326.00)	104.00 (39.00, 417.0)	144.00 (65.00, 264.00)	**0.03**
**Glucose (mg/dL)**	90.00 (76.00, 314.00)	91.00 (70.00, 141.00)	96.00 (77.00, 111.00)	0.50
**Insulin (U/mL)**	12.50 (3.20, 4.20)	13.20 (3.70, 36.00)	10.90 (4.70, 64.30)	0.80
**HOMA-IR**	2.92 (0.63, 18.06)	3.20 (0.60, 8.64)	2.60 (0.93, 17.31)	0.88
**QUICKI**	0.32 0.03	0.32 0.02	0.32 0.03	**0.97**
**AgRP (pg/mL)**	24.80 (12.60, 90.00)	23.70 (14.70, 94.00)	24.10 (15.20, 78.00)	0.57
**α-MSH (ng/mL)**	145.75 (109.6, 678.0)	147.5 (95.10, 689.0)	142.0 (106.1, 665.0)	0.56

*BMI* Body Mass Index, *WC* Waist Circumference, *WHR* Waist-to-Hip Ratio, *FM* Fat Mass, *BMR* Basal Metabolic Rate, *PA* Physical Activity, *SBP* Systolic Blood Pressure, *DBP* Diastolic Blood Pressure; values for gender is in number of subjects (percentage) and other data are presented based on mead (SD) or median (min, max).^*^P values based on One-Way ANOVA; Bold values present the statistically significant threshold

**Table 3:** Odd’s ratio (OR) and confidence interval (CI) for the association between metabolic factors and FADS2 rs174583 genotypes

	TT	CT	CC
**Weight** (kg)			
**Crude**	1(Ref.)	1.13 (0.68-1.87)	1.01 (0.58-1.78)
**Model 1**^a^	1(Ref.)	1.12 (0.71-1.78)	1.01 (0.60-1.68)
**FM (%)**			
**Crude**	1(Ref.)	0.87 (0.52-1.47)	0.96 (0.54-1.70)
**Model 1**	1(Ref.)	0.85 (0.53-1.36)	0.93 (0.55-1.57)
**FFM (%)**			
**Crude**	1(Ref.)	0.87 (0.52-1.45)	0.95 (0.54-1.67)
**Model 1**	1(Ref.)	0.85 (0.54-1.36)	0.95 (0.56-1.59)
**WC (cm)**			
**Crude**	1(Ref.)	2.06 (0.57-7.45)	1.52 (0.40-5.82)
**Model 1**	1(Ref.)	2.12 (0.53-8.37)	1.48 (0.30-6.26)
**BMI** (kg/m^2^)			
**Crude**	1(Ref.)	1.13 (0.72-1.75)	1.08 (0.69-1.70)
**Model 1**	1(Ref.)	1.19 (0.76-1.87)	1.13 (0.71-1.80)
**HC (cm)**			
**Crude**	1(Ref.)	0.46 (0.13-1.59)	0.65 (0.18-2.38)
**Model 1**	1(Ref.)	0.45 (0.12-1.70)	0.67 (0.16-2.70)
**SBP** (mmHg)			
**Crude**	1(Ref.)	1.01 (0.94-1.08)	0.97 (0.91-1.05)
**Model 1**	1(Ref.)	1.01 (0.93-1.10)	0.98 (0.90-1.09)
**DBP** (mmHg)			
**Crude**	1(Ref.)	1.01 (0.93-1.11)	1.04 (0.95-1.13)
**Model 1**	1(Ref.)	1.03 (0.94-1.13)	1.05 (0.96-1.16)
**Glucose (mg/dL)**			
**Crude**	1(Ref.)	0.99 (0.92-1.06)	1.005 (0.93-1.08)
**Model 1**^a^	1(Ref.)	1.003 (0.92-1.08)	1.02 (0.94-1.10)
**TC (mg/dL)**			
**Crude**	1(Ref.)	0.99 (0.97-1.01)	0.98 (0.96-1.009)
**Model 1**	1(Ref.)	0.98 (0.96-1.01)	0.98 (0.96-1.007)
**TG (mg/dL)**			
**Crude**	1(Ref.)	0.99 (0.98-1.005)	0.99 (0.97-1.004)
**Model 1**	1(Ref.)	0.99 (0.97-1.005)	0.98 (0.97-1.003)
**HDL (mg/dL)**			
**Crude**	1(Ref.)	**1.12 (1.01-1.25)**	**1.11 (1.00-1.23)**
**Model 1**	1(Ref.)	**1.14 (1.01-1.28)**	**1.13 (1.007-1.27)**
**Insulin (U/mL)**			
**Crude**	1(Ref.)	0.79 (0.52-1.21)	0.85 (0.55-1.32)
**Model 1**	1(Ref.)	0.80 (0.52-1.22)	0.88 (0.56-1.38)
**HOMA-IR**			
**Crude**	1(Ref.)	1.27 (0.29-5.42)	1.27 (0.28-5.68)
**Model 1**	1(Ref.)	1.21 (0.28-5.25)	1.13 (0.24-5.26)
**α-MSH (ng/L)**			
**Crude**	1(Ref.)	1.00 (0.99-1.01)	1.00 (0.99-1.01)
**Model 1**	1(Ref.)	1.00 (0.99-1.01)	1.003 (0.99-1.01)
**Ag-RP (Pg/ml)**			
**Crude**	1(Ref.)	0.98 (0.91-1.07)	0.98 (0.90-1.07)
**Model 1**	1(Ref.)	0.98 (0.90-1.07)	0.97 (0.89-1.06)

The multivariate multinomial logistic regression was used for estimation of ORs and confidence interval (CI). ^a^Adjusted for age, sex, physical activity and socio-economic status; Bold values present the statistically significant threshold

## Discussion

To the best of our knowledge, this is the first study to investigate the effects of the interactions between FADS2 gene rs174583 polymorphism and two dietary indices (DQI-I and HEI) on cardiometabolic risk factors, including serum lipids, markers of glucose hemostasis, blood pressure, AgRP, and α-MSH in obese individuals. According to our results, it seems that while obese individuals with TT homozygote genotype are more prone to have higher levels of weight, BMI, WC, FM, TC, LDL, TG, and insulin, they have lower HDL levels. Similarly, Mazoochian et al. evaluated the effect of FADS2 gene rs174583 polymorphism on biochemical parameters among T2D patients in Iran and reported the highest levels of TC and TG among homozygotes for T alleles, and the difference was significant in term of TG. Also, in line with our findings, they illustrated that the lowest level of HDL belonged to TT carriers [29]. Moreover, a study in South Korea showed a significant difference in terms of TG and glucose across FADS2 genotypes, but other metabolic parameters including WC, HDL, SBP, and DBP did not differ significantly. Also, they reported an association between FADS2 genotypes and cardiometabolic components [45]. Our results were also in agreement with another study conducted among Japanese males, reporting higher levels of TG in homozygotes for T allele of FADS2 gene rs174583 polymorphism [46].

In our study, FADS2 gene-diet interactions affected some anthropometric and glycemic markers with no significant interaction in terms of lipid profile. It is expected that high adherence to DQI-I and HEI with higher intake of EPA and DHA may contribute to reduced level of TG through increased β-oxidation and clearance of TG-rich lipoproteins; while gene variants have affected the level of serum lipids in response to dietary intervention [47]. Interestingly, Abu-Mweis et al. illustrated more beneficial effects of DHA combined with high-oleic canola oil supplementation on TG levels in comparison to canola oil supplemented group with heterozygous CT genotypes; however, the difference was not statistically significant [47]. Although the underling mechanism for the effect of FADS2 gene variants on HDL concentration remains unclear, it is suggested that its gene variants may impair desaturase function contributing to low level of n-6 and n-3 production. PUFAs have been introduced as a ligand for peroxisome proliferator activating receptor-α and are involved in the regulation of lipoprotein lipase and apo-lipoprotein A-I, A-II, and C-III, modulating HDL metabolism [15, 48, 49]; these discrepancies may be attributed to the study population and dietary patterns and habits. Furthermore, Warensjo et al. indicated that D6D and triacylglycerol in adipose tissue were correlated with BMI and insulin resistance [50]. Indeed, desaturase enzymes are incorporated in the synthesis of LC-PUFAs, which are involved in cell membrane affecting permeability of cells. This may affect insulin signaling and receptor binding affinities. In other words, the products of D6D have been proposed as a ligand for transcription factors, such as sterol regulatory element binding protein 1 (SREBP-1) and peroxisome proliferators activated receptors, and they may interact with genes involved in lipogenesis and fatty acid oxidation subsequently [15, 48, 49].

Despite the higher levels of weight, BMI, and FM in homozygote TT genotypes, moderate adherence to DQI-I improved the level of these values and attenuated the adverse effects of risk allele. Similarly, the highest levels of glycemic markers, including insulin, HOMA-IR, QUICKI, and α-MSH were reported among TT carriers. Nevertheless, high compliance with HEI could not modify the adverse effects of having TT genotype on insulin and HOMA-IR. On the other hand, high adherence to HEI exerted its favorable effect on heterogeneous genotype in terms of insulin and HOMA-IR. In contrast, higher adherence to HEI had ameliorative effects on QUICKI and α-MSH even in TT carriers after adjusting for potential confounders. Evidence shows that HOMA-IR is related to fatty acid composition (LA and DGLA). Kim et al. [51] found an interactive effect of rs174575 SNP of FADS2 and the proportion of DGLA or AA on HOMA-IR levels. In addition, previous studies indicated the relationship between serum phospholipid fatty acid composition or dietary fat with insulin resistance, reporting higher levels of palmitic acid in people with insulin resistance, which was also confirmed in another study by Warensjo et al. [50]. Positive relationships have also been reported between obesity and insulin resistance and palmitoleic acid and DGLA in serum phospholipids [50, 51].

It is worth noting that despite ever-increasing knowledge in this field, there is no study to evaluate the effect of interactions between FADS2 gene polymorphism and dietary indices on cardiometabolic risk factors. The present study focused on the modifiable effects of dietary indices (DQI-I and HEI) on obesity outcomes in different FADS2 genotypes with different susceptibility to risk factors among a relatively acceptable sample size, which provides an effective and dynamic nutritional approach to design tailored nutrition recommendations. However, the cross-sectional design of the study limits understanding the causal relationships. Therefore, further longitudinal studies are necessary to confirm our findings.

## Conclusion

This study, for the first time, evaluated the effects of the interactions between FADS2 gene rs174583 polymorphism and two dietary indices (DQI-I and HEI) on cardiometabolic risk factors. According to the results, compliance with these dietary indices ameliorated the adverse effects of gene variants on metabolic risk factors, especially in heterogeneous genotypes. Further prospective cohort studies are needed to confirm these results.

## Supplementary Information

**Additional file 1: Supplementary Table 1.** Comparison of dietary vitamins between FADS2 gene polymorphism. **Supplementary Table 2.** Representing the interactions between FADS2 gene polymorphism and DQI-I in term of metabolic factors. **Supplementary Table 3.** Representing the interactions between FADS2 gene polymorphism and HEI in term of metabolic factors.

## Data Availability

The author have some restrictions from the Ethics Committee of Tabriz University of Medical Sciences for sharing data of the current study. All the data are available with reasonable request from the corresponding author.

## Abbreviations

T2DM: Type two diabetes mellitus
BMI: Body mass index
GWAS: Genome wide association studies
SNP: Single nucleotide polymorphisms
FADS2: Fatty acid desaturase-2
D6D: Delta 6 desaturase
LC-PUFAs: Long chain poly unsaturated fatty acids
LA: Linoleic acid
α-ALA: Alpha linolenic acid
DGLA: Dihomo gamma-linoleic acid
AA: Arachidonic acid
EPA: Ecosapantanoic acid
DQI-I: Diet quality index-International
HEI: Healthy eating index
SES: Socioeconomic status
IPAQ: International Physical Activity Questionnaire
HC: Hip circumference
BIA: Bioelectrical impedance analysis
SBP: Systolic blood pressure
DBP: Diastolic blood pressure
TG: Triglyceride
TC: Total cholesterol
HDL-C: High-density lipoprotein cholesterol
LDL-C: Low-density lipoprotein cholesterol
HOMA-IR: Homeostasis model assessment-insulin resistance index
QUICKI: Quantitative insulin sensitivity check index
Ag-RP: Agouti-related peptide
α-MSH: α-melanocyte-stimulating hormone
VAS: Visual analogue scale
FFQ: Food frequency questionnaire
FCT: Food composition table
PCR-RFLP: Polymerase chain reaction-restricted length polymorphism
SREBP-1: Sterol regulatory element binding protein 1
