# Supplementary material for: The integrative panel of fatty acid desaturase-2 (FADS2) rs174583 gene polymorphism and dietary indices (DQI-I and HEI) affects cardiovascular risk factors among obese individuals
Source: BMC Endocr Disord. 2023 Feb 15;23:41. doi: 10.1186/s12902-023-01289-3 (PMC9930302; doi:10.1186/s12902-023-01289-3)
Supplement: Supplementary file 1 — Additional file 1: Supplementary Table 1. Comparison of dietary vitamins between FADS2 gene polymorphism. Supplementary Table 2. Representing the interactions between FADS2 gene polymorphism and DQI-I in term of metabolic factors. Supplementary Table 3. Representing the interactions between FADS2 gene polymorphism and HEI in term of metabolic factors. [file 12902_2023_1289_MOESM1_ESM.docx]

**Title: The integrative panel of fatty acid desaturase-2 (FADS2) rs174583 gene polymorphism and dietary indices (DQI-I and HEI) affects cardiovascular risk factors among obese individuals**

**Authors: Mahsa Mahmoudinezhad ^1^, Goli Siri ^2^, Leila Saljoughi ^3^, Mahdieh Abbasalizad Farhangi ^4*^, Houman Kahroba ^5, 6^**

^1^ Molecular Medicine Research Center, Tabriz University of Medical Sciences, Tabriz, Iran

^2^ Department of Internal Medicine, Amir-Alam Hospital, Tehran University of Medical Sciences, Tehran, Iran

^3^ Department of Internal Medicine, School of Medicine, Shahid Beheshti University of Medical Sciences, Tehran, Iran

^4^ Drug Applied Research Center, Tabriz University of Medical Sciences, Tabriz, Iran.

^5^ Department of Toxicogenomics, GROW School of Oncology and Development Biology, Maastricht University, the Netherlands.

^6^ Centre for Environmental Sciences, Hasselt University, Hasselt, Belgium.

Corresponding author E-mail: [abbasalizad_m@yahoo.com](mailto:abbasalizad_m@yahoo.com). Postal code: 5165665931

Attar-neishabouri Ave, Golgasht St, Tabriz, Iran. Phone: +04133357584

**Supplementary Material**


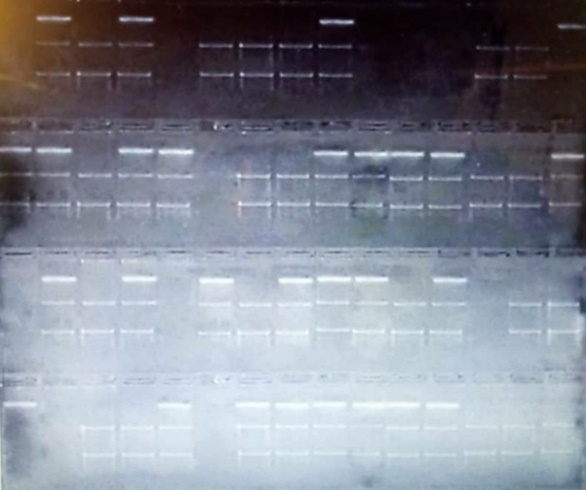

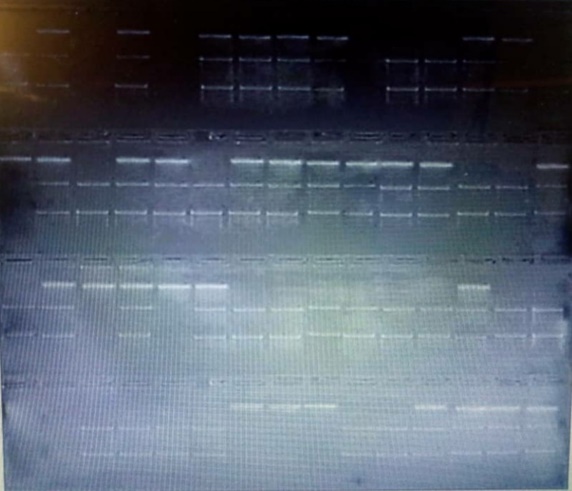

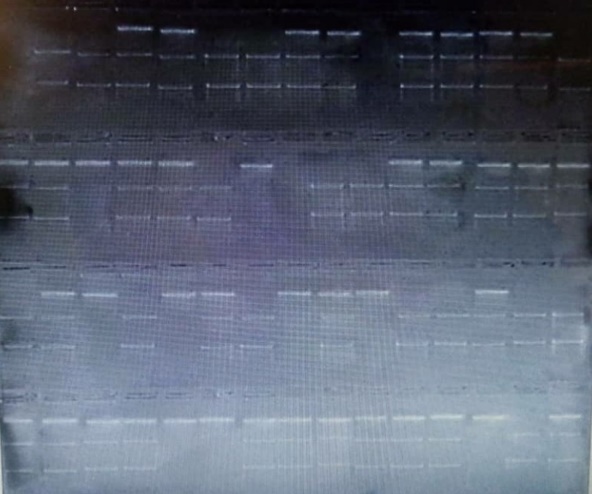

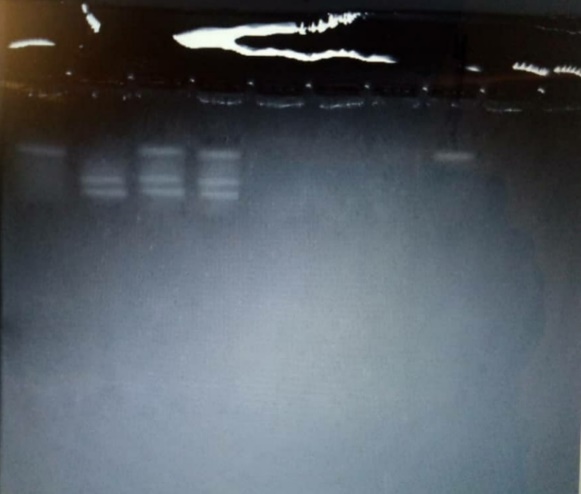


**Supplementary Figure 1.** PCR analysis for rs174583 gene polymorphism.

**Supplementary Table 1.** Comparison of dietary vitamins between FADS2 gene polymorphism.

|  | **CC** | **CT** | **TT** | **P-value** |
| --- | --- | --- | --- | --- |
| **Vitamin A (RAE)** | 1005.35 ± 907.95 | 906.44 ± 715.30 | 1196.69 ± 1143.24 | 0.42 |
| **Vitamin D (mg)** | 2.01 ± 1.38 | 2.17 ± 1.59 | 2.95 ± 2.63 | 0.13 |
| **Vitamin E (mg)** | 19.06 ± 11.80 | 16.53 ± 8.89 | 18.78 ± 8.70 | 0.31 |
| **Vitamin K** | 304.41 ± 266.29 | 283.12 ± 327.38 | 250.62 ±184.51 | 0.79 |
| **Thiamin (mg)** | 2.70 ± 1.22 | 2.56 ± 1.06 | 3.04 ± 1.46 | 0.31 |
| **Riboflavin (mg)** | 2.56 ± 1.20 | 2.50 ± 0.94 | 3.06 ± 1.09 | 0.15 |
| **Niacin (mg)** | 29.80 ± 13.08 | 27.97 ± 10.04 | 34.41 ± 13.76 | 0.12 |
| **Pantothenic (mg)** | 6.48 ± 2.54 | 6.26 ± 2.11 | 7.66 ± 2.46 | 0.09 |
| **Vitamin B6 (mg)** | 2.31 ± 1.21 | 2.07 ± 0.66 | 2.44 ± 0.73 | 0.19 |
| **Folate (mg)** | 772.62 ± 379.39 | 702.52 ± 249.00 | 876.39 ± 487.82 | 0.12 |
| **Folat.DFE (mg)** | 878.89 ± 458.27 | 818.24 ± 327.02 | 1040.47 ± 605.18 | 0.14 |
| **Vitamin B12 (mg)** | 5.87 ± 8.20 | 5.58 ± 6.68 | 9.37 ± 11.64 | 0.24 |
| **Biotin (mg)** | 40.57 ± 21.92 | 37.59 ± 15.39 | 44.16 ± 14.90 | 0.34 |

BMI, Body Mass Index; WC, Waist Circumference; WHR, Waist-to-Hip Ratio; FM, Fat Mass; BMR, Basal Metabolic Rate; PA, Physical Activity; SBP, Systolic Blood Pressure; DBP, Diastolic Blood Pressure; values for gender is in number of subjects (percentage) and other data are presented based on mead (SD) or median (min, max).*^*^* P values based on One-Way ANOVA.

**Supplementary Table 2.** Representing the interactions between FADS2 gene polymorphism and DQI-I in term of metabolic factors.

|  | **P-interaction** |
| --- | --- |
| Weight (kg) | **0.01** |
| FM (%) | **0.04** |
| FFM (%) | **0.03** |
| WC (cm) | 0.47 |
| HC (cm) | **0.005** |
| BMI (kg/m2) | **0.02** |
| WHR | 0.1 |
| SBP (mmHg) | 0.64 |
| DBP (mmHg) | 0.94 |
| Glucose (mg/dL) | 0.61 |
| TC (mg/dL) | 0.17 |
| TG (mg/dL) | 0.9 |
| HDL (mg/dL) | 0.68 |
| LDL (mg/dL) | 0.16 |
| Insulin (U/mL) | 0.21 |
| HOMA-IR | 0.12 |
| QUICKI | 0.36 |
| α-MSH (ng/L) | 0.42 |
| Ag-RP (Pg/ml) | 0.15 |

**Supplementary Table 3.** Representing the interactions between FADS2 gene polymorphism and HEI in term of metabolic factors.

|  | **P-interaction** |
| --- | --- |
| Weight (kg) | 0.06 |
| FM | 0.29 |
| FFM | 0.45 |
| WC | 0.35 |
| HC | 0.41 |
| BMI | 0.70 |
| WHR | 0.26 |
| SBP | 0.34 |
| DBP | 0.62 |
| Glucose | 0.19 |
| Cholesterol | 0.07 |
| Triglyceride | 0.64 |
| HDL | 0.22 |
| LDL | 0.11 |
| Insulin | **<0.001** |
| HOMA-IR | **<0.001** |
| QUICKI | **0.01** |
| α-MSH (ng/L) | **0.03** |
| Ag-RP (Pg/ml) | 0.05 |
